# Facial Spasm Caused by Compression of the Distal Portion of the Facial Nerve by the Anterior Inferior Cerebellar Artery, Resulting in Delayed Peripheral Facial Nerve Palsy: A Case Report

**DOI:** 10.7759/cureus.39311

**Published:** 2023-05-21

**Authors:** Seigo Kimura, Ryokichi Yagi, Keiichi Yamada, Hirokatsu Taniguchi, Masahiko Wanibuchi

**Affiliations:** 1 Neurosurgery, Kouzenkai Yagi Neurosurgical Hospital, Osaka, JPN; 2 Neurosurgery, Osaka Medical and Pharmaceutical University, Takatsuki, JPN

**Keywords:** distal portion of the facial nerve, anterior inferior cerebellar artery, microvascular decompression, delayed facial nerve palsy, facial spasm

## Abstract

Delayed onset of facial spasm following surgery for unilateral facial spasm after microvascular decompression (MVD) of the distal facial nerve is rare. We report a case of unilateral facial spasm caused by compression of the distal facial nerve successfully treated with MVD resulting in delayed peripheral facial nerve palsy. A 51-year-old male patient with a left facial spasm showed a gradual worsening of symptoms. Head imaging revealed that the left anterior inferior cerebellar artery (AICA) was in contact with the distal portion of the left facial nerve; hence, MVD was performed. The AICA was pressing on the distal facial nerve, so the AICA was transpositioned. Postoperatively, the facial spasm improved. On the eighth postoperative day, the patient showed left peripheral facial nerve palsy and was given conservative treatment. The patient was discharged home on the sixteenth postoperative day. One month after discharge, the facial palsy was in complete remission. Distal facial nerve compression may cause unilateral facial spasms. Although delayed facial nerve palsy may occur, the prognosis is good.

## Introduction

Unilateral facial spasm is a syndrome caused by direct vascular compression of the facial nerve, and facial nerve compression is commonly observed in the root exit zone (REZ). Unilateral facial spasm caused by vascular compression of the distal facial nerve is rare [1]. Microvascular decompression (MVD) has been used as a surgical treatment for unilateral facial spasms; however, there have been some reports of delayed postoperative facial nerve palsy [2]. Further, delayed onset of facial spasm following surgery for unilateral facial spasm after MVD of the distal facial nerve is extremely rare. Herein, we report a case of unilateral facial spasm caused by medial compression of the distal facial nerve by the anterior inferior cerebellar artery (AICA) treated with MVD, resulting in delayed peripheral facial nerve palsy.

## Case presentation

**Figure 1:**
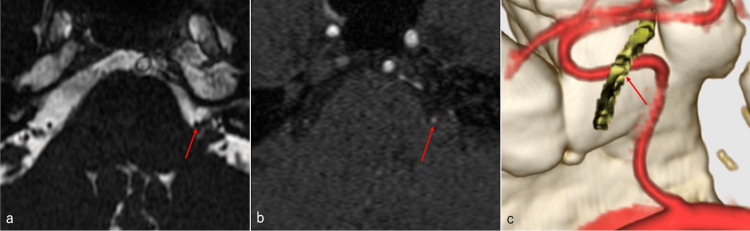
Left anterior inferior cerebellar artery in contact with the distal portion of the left facial nerve The left anterior inferior cerebellar artery was found to be in contact with the distal portion of the left facial nerve, but no responsible vessel could be noted in the root exit zone of the left facial nerve. a) Head magnetic resonance imaging (fast imaging employing steady-state acquisition); b) Head magnetic resonance angiography original image; c) 3D structured image. Red arrow: Distal facial nerve compression site by the left anterior inferior cerebellar artery.

A 51-year-old male patient had a left facial spasm for eight years. Head magnetic resonance imaging (MRI) performed at another hospital indicated left facial nerve compression owing to left AICA. Initially, he experienced mild symptoms and was followed up, but his symptoms gradually worsened. The patient did not want to receive treatment with antiepileptic drugs and hence visited our hospital for surgery. There was no relevant medical history. On admission, the patient was conscious with a left facial spasm, and no other obvious neurological abnormalities were found. The head MRI (fast imaging employing steady-state acquisition: FIESTA) and head magnetic resonance angiography original images, as well as 3D structural images (created by the volume analyzer, SYNAPSE VINCENT (Fujifilm, Tokyo, Japan) using the head computed tomography angiography and MRI (FIESTA)), indicated that the left AICA was in contact with the distal portion of the left facial nerve. However, the responsible vessel could not be identified in the REZ of the left facial nerve (Figure 1).

**Figure 2:**
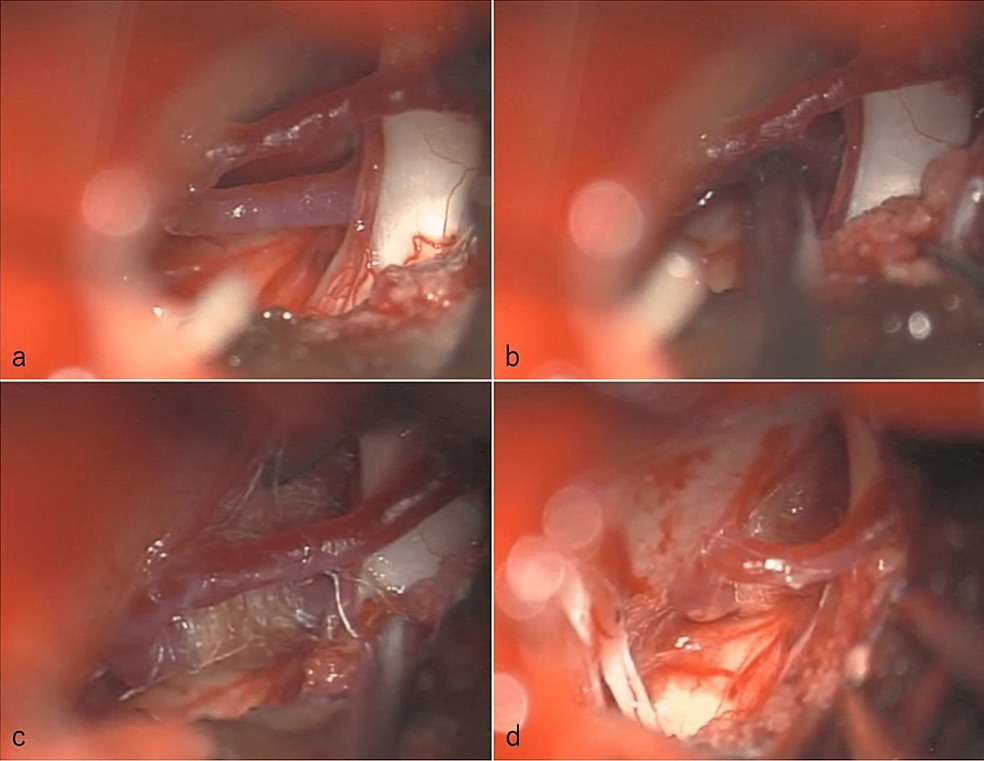
Microvascular decompression of the distal facial nerve by the anterior inferior cerebellar artery a) Medial compression of the distal facial nerve by the anterior inferior cerebellar artery was observed. b) When the abnormal muscle response was checked with the anterior inferior cerebellar artery separated from the facial nerve, the abnormal waveform that had appeared until then disappeared. c) Anterior inferior cerebellar artery was transpositioned using Teflon tape and fixed to the clivus. d) Final image.

The patient actually had a unilateral facial spasm, and distal left facial nerve compression by the AICA was thought to be the responsible lesion for the unilateral facial spasm. Hence, MVD was performed with informed consent. Auditory brainstem response (ABR) and abnormal muscle response (AMR) were used for monitoring. The patient was operated on using a lateral suboccipital approach. After craniotomy, a U-shaped dural incision was made. Then, the lateral medullary cistern was opened. The AICA was medially pressing on the distal facial nerve but did not touch the REZ (Figure 2a). No other vessels compressing the facial nerve or REZ were noted. On checking the AMR after separating the AICA from the facial nerve, the abnormal waveform that appeared previously disappeared. Thus, it was established that the lesion responsible for the left facial spasm was AICA (Figure 2b). The AICA was transpositioned with Teflon tape and placed on the clivus (Figure 2c). After adequate fixation, AMR was performed again to confirm the disappearance of abnormal waveforms (Figure 2d). During surgery, ABR showed no abnormalities. The wound was closed as before, and the surgery was completed.

**Figure 3:**
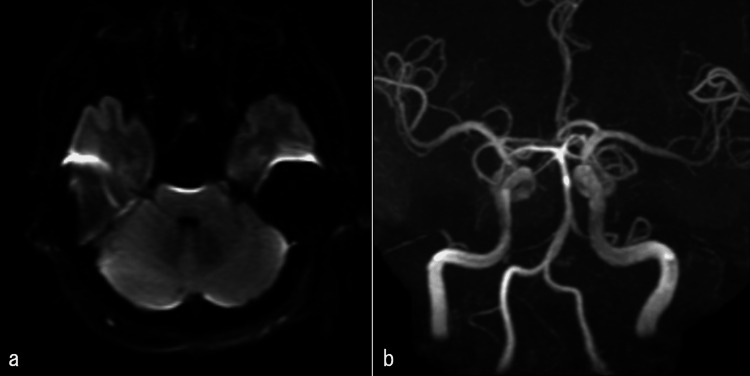
Head magnetic resonance imaging and angiography on the next day of the surgery a) Head magnetic resonance imaging (diffusion-weighted imaging): No obvious acute ischemia. b) Head magnetic resonance angiography: No obvious vascular occlusion.

Postoperatively, the left facial spasm improved. Furthermore, a head MRI performed on the day after surgery showed no obvious cerebral infarction or vascular occlusion (Figure 3).

**Figure 4:**
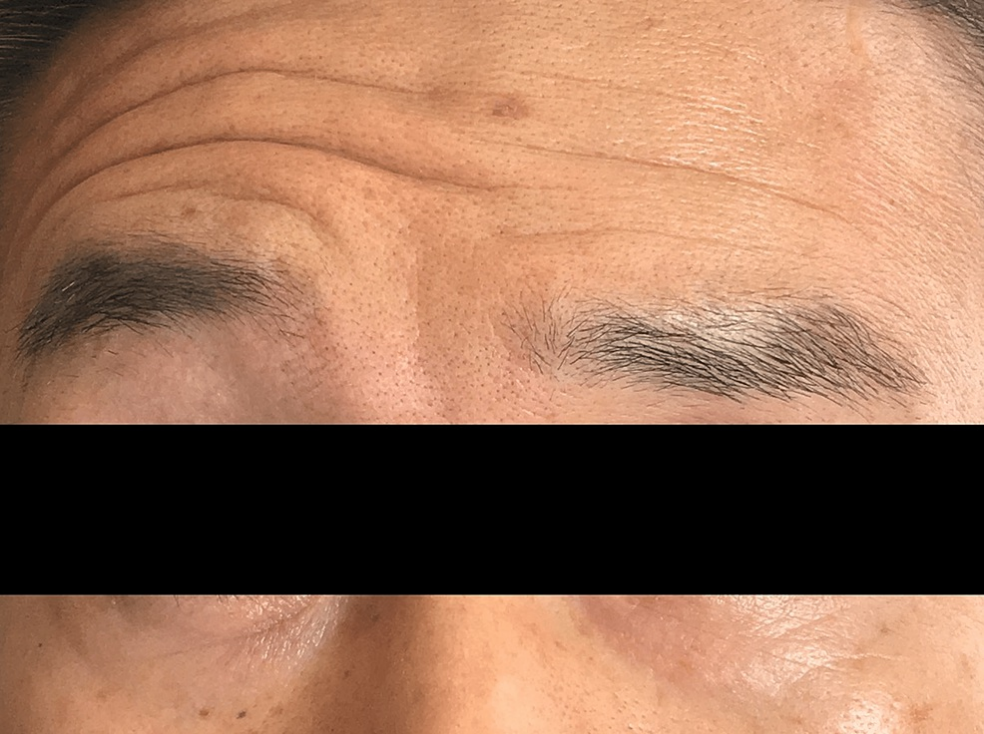
Delayed-onset left peripheral facial nerve palsy On the eighth postoperative day, the patient was unable to wrinkle the left forehead. A diagnosis of delayed-onset left peripheral facial nerve palsy (House–Brackmann grade III) was made.

The patient had no significant postoperative abnormality. But, on the eighth postoperative day, the patient was unable to wrinkle the left forehead and experienced difficulty closing the left eye. Additionally, the left angle of his mouth was droopy (Figure 4).

A diagnosis of delayed-onset left peripheral facial nerve palsy (House-Brackmann grade III) was made, and prednisolone 30 mg/day, methylcobalamin 1500 μg/day, and valaciclovir 1000 mg/day were administered. On the sixteenth postoperative day, the patient was discharged home. Prednisolone was tapered off. Peripheral facial palsy was followed up at an outpatient clinic. One month after discharge, the peripheral facial palsy was in complete remission, and the left facial spasm had not recurred.

## Discussion

A unilateral facial spasm is usually caused either by an AICA forming a caudal loop or a posterior inferior cerebellar artery (PICA) forming an upward loop that contacts the REZ [3]. However, in our case, the AICA forming a caudal loop ran medial to the facial nerve and compressed the distal facial nerve from the medial side. A unilateral facial spasm occurs mainly because of the vascular compression of the REZ. However, there are scattered reports of onset due to distal facial nerve compression. In previous reports, the following four patterns of compression vessels in the distal facial nerve were observed: 1) double compression in the distal facial nerve as well as the REZ; 2) a compression vessel between the facial and auditory nerves; 3) a compression vessel in the distal facial nerve at reoperation; and 4) a single compression vessel in the distal facial nerve, except between the facial and auditory nerves at initial surgery [1]. Among these, to the best of our knowledge, only 13 cases with patients with AICA as the compression vessel: one of 142 patients by Ryu et al. [4], three of 115 patients by Campos-Benitez et al. [5], eight of 2137 patients by Chang et al. [6], and one of two patients by Son et al. [1]. The oligodendroglia making up the myelin sheath of the facial nerve axon in the brainstem are replaced by Schwann cells in the REZ [7]. Unilateral facial spasms are less likely to occur with peripheral vascular compression of the facial nerve because Schwann cells are resistant to vascular compression that causes facial nerve demyelination [8]. The distal facial nerve is longer than the central facial nerve; the latter is less organized than the distal facial nerve and is considered more vulnerable to physical compression due to its thinner myelin, structural characteristics, and lack of undulation of nerve fibers [9,10]. It has been considered that more severe physical compression of the peripheral facial nerve myelinated by Schwann cells is required to cause facial spasms [6]. Notably, unilateral facial spasms can develop at sites other than the REZ if severe vascular compression of the distal facial nerve is present.

Chang et al. reported that the clinical outcome of patients with distal facial nerve compression was worse than that of patients with proximal compression and cited two reasons for the poor outcome: 1) severe structural and physiological changes occurring in the facial nerve because of severe physical compression of the distal facial nerve and 2) diagnostic inaccuracies [6]. There are few reports on the effectiveness of AMR in MVD for distal facial nerve compression as a technique to compensate for diagnostic inaccuracies; in cases of distal facial nerve compression by AICA, abnormal waveforms in AMR disappeared when AICA was decompressed and recurred when AICA resurfaced [11,12]. In our case, the disappearance of the abnormal waveform on AMR allowed us to determine the responsible lesion. Furthermore, the left facial spasm improved after surgery. The vascular compression of the distal facial nerve can also cause unilateral facial spasms. Hence, AMR is considered to be a useful monitoring tool for surgery.

Lovely et al. reported delayed facial nerve palsy to be a rare complication after performing MVD for unilateral facial spasms with REZ compression [2]. According to that report, it occurred in 2.8% of patients with an average time to onset of 12 days, and the cause was unknown. The patients had a good prognosis with spontaneous remission in almost all cases. To the best of our knowledge, there were no case reports of delayed facial nerve palsy after MVD in patients with facial spasms due to distal facial nerve compression, and we believe we were the first to report this. In our patient, a House-Brackmann grade III left delayed facial nerve palsy occurred eight days after MVD. No facial nerve palsy was observed immediately after surgery until the seventh postoperative day, making it difficult to believe that the surgical technique affected the patient. Lovely et al. discussed that the consistent timing of the delayed peripheral facial nerve palsy does suggest the involvement of a virus lying dormant in the geniculate ganglion, which is triggered by manipulation of the nerve or nervus intermedius [2]. A similar mechanism may be relevant in our case. The patient’s symptoms improved with conservative treatment, including steroid administration, and he made good progress. Delayed facial nerve palsy after MVD surgery for unilateral facial spasm caused by compression of the distal portion of the facial nerve was thought to follow a similarly favorable course as delayed facial nerve palsy that develops after MVD for unilateral facial spasm with REZ compression.

## Conclusions

We report a case of unilateral facial spasm caused by medial compression of the distal facial nerve by AICA treated with MVD, resulting in delayed peripheral facial nerve palsy. Compression of the distal facial nerve may cause unilateral facial spasms. AMR is useful in MVD due to distal facial nerve compression. Delayed facial nerve palsy may occur, but spontaneous remission is also possible, and the prognosis is good.
